# Social determinants of health, data science, and decision-making: Forging a transdisciplinary synthesis

**DOI:** 10.1371/journal.pmed.1003174

**Published:** 2020-06-11

**Authors:** Sandro Galea, Salma M. Abdalla, Jeffrey L. Sturchio

**Affiliations:** 1 School of Public Health, Boston University, Boston, Massachusetts, United States of America; 2 Rabin Martin, New York, New York, United States of America

## Abstract

Sandro Galea and co-authors discuss a forthcoming Collection on data science and social determinants of health.

Two parallel, intellectual strands that seldom intersect have emerged in our evolving understanding of health and health systems over the past couple of decades. First is the growing appreciation of the role that social and economic factors play in shaping health. These so-called social determinants of health are now widely accepted to be core contributors to the health of populations, as indicated by a substantial body of empirical work. We know, for example, that income and education fundamentally shape health through the life course, that disparities by sex and ethnicity affect health outcomes, that characteristics of place (including the built environment) determine opportunities for health-promoting behavior and access to healthcare resources, and that public policies, market incentives, and commercial forces are inextricably linked with the conditions that ultimately generate health [1–4]. This has led to recognition of the centrality of these concepts by public health scholars and governmental organizations at all levels. The 2008 report of WHO Commission on Social Determinants of Health, chaired by Michael Marmot, was a landmark study in this area [5]. The United States Department of Health and Human Services has advocated for Public Health 3.0, which urges thinking beyond clinical settings to include complementary perspectives from community institutions to link prevention and treatment with social, economic, and environmental factors that help explain patterns of disease in given populations. Several books have translated these ideas for the general public [6,7], and slowly, the political- and private-sector conversation is also beginning to recognize the role that social determinants play in health.

A second strand has followed the emergence of technologies that can collect data that characterize our biology, how we live, and the forces with which we intersect with far greater granularity than was ever possible before. These “big data” approaches (characterized typically by large volumes of data, collected quickly, and varied across levels of human organization [e.g., from the biological to the social]) have paved the way for unprecedented, new opportunities for measuring, analyzing, and documenting individual and population health. In particular, these approaches (enabled by new data-analytic tools from artificial intelligence and machine learning) have deployed new data sources and new technologies that can improve health and improve decision-making at the individual and community level. The potential of big data to improve health is becoming apparent both in the genomic revolution that has created an enormous growth in precision medicine approaches to health and in the digital revolution more broadly. These technological advances have made it possible to obtain more data about aspects of human activity, behavior, and context. Recent developments in genomics, big data, and artificial intelligence offer intriguing prospects for the development of a new discipline of precision public health, which promises to deliver greater improvements in population health outcomes by providing “the right intervention to the right population at the right time” [8].

These two conceptual agendas have developed separately through different communities with relatively little intersection. It is not difficult to see, however, how each can inform the other. The understanding that social and economic conditions influence health suggests that better surveillance of these same determinants can guide opportunities for interventions designed to improve health in populations. Similarly, recognizing that health is ineluctably linked to these same exogenous factors can nudge data science to collect behavioral, network, and community data that can both contextualize and more fully inform our understanding of the biological mechanisms that ultimately manifest as disease. This can, in turn, lead to better evidence-informed decisions—by policymakers at global, national, and community levels—whether in multilateral organizations, national governments, local communities, corporations, healthcare purchasers, or provider institutions—to improve individual and population health.

This observation leads us to suggest that it is time to bring about a transdisciplinary synthesis of these different streams. The social determinants of health represent an agenda for action to improve health that must encompass factors such as the built and social environments of neighborhoods and government policies relevant to health. This agenda can be enhanced by a serious engagement with the role that data and technology are beginning to play in improving population health. We have seen some early efforts, such as the aforementioned Public Health 3.0 initiative that has seen progress on population health as a “convergence science” [9]. In addition, in the US, an evolving Surgeon General’s report aims to understand how thinking about social determinants can bring about better health in communities [10]. The Pan American Health Organization emphasizes the need for intersectoral action on social determinants of health to achieve universal access to health [11]. These efforts point to the observation that progress in population health cannot depend on a single sector and requires scientific understanding of education, social services, economic development, environment (both built and natural), nutrition and food marketing, urban design, and health. Success, seen through this lens, depends on effective partnerships across sectors that can better inform understanding of these areas, bring to them improved measurement and surveillance, and develop innovative approaches to have this understanding inform the work of decision makers.

Aiming to accelerate this synthesis, we have recently launched the Rockefeller Foundation-Boston University Commission on Health Determinants, Data, and Decision-Making (the 3-D Commission; see Acknowledgments). The 3-D Commission brings experts from both the fields of social determinants and data science together with decision makers. The Commission aims both to improve our understanding of the forces that shape health, and also to create recommendations that will inform decision-making to improve the health of the global population. The project builds on the observation that social and economic determinants matter, that data can help us understand how they matter, and used together to inform decision-making, will help us improve population health.

At the core, the project asks two fundamental questions. First, how do we create a new data-driven approach to inform guidance for policymakers and practitioners who aim to improve health through improving the social and economic conditions that drive health both globally and locally? Second, how do we create a demand for public and private investment in the social and economic determinants of health?

Importantly, while seeking to engage the literature to map the opportunities emerging from social determinants of health and data, this Commission is focused on creating pragmatic and action-oriented recommendations to support decision makers when designing policies and interventions to improve the health of populations. We will be publishing a report that elaborates on these ideas and a collection of papers that advance our thinking in the area. Our hope is that this work will inform action in this area in coming years toward the goal of establishing a more integrated and powerful approach to improving the health of populations efficiently and sustainably.
